# Cannabidiol Inhibits the Proliferation and Invasiveness of Prostate Cancer Cells

**DOI:** 10.1021/acs.jnatprod.3c00363

**Published:** 2023-09-13

**Authors:** Eve O’Reilly, Karima Khalifa, Joanne Cosgrave, Haleema Azam, Maria Prencipe, Jeremy C. Simpson, William M. Gallagher, Antoinette S. Perry

**Affiliations:** § UCD School of Biology and Environmental Science, University College Dublin, Dublin D04 C1P1, Ireland; † Cancer Biology and Therapeutics Laboratory, Conway Institute of Biomolecular and Biomedical Research, University College Dublin, Dublin D04 C1P1, Ireland; ‡ UCD School of Biomolecular and Biomedical Science, University College Dublin, Dublin D04 C1P1, Ireland

## Abstract

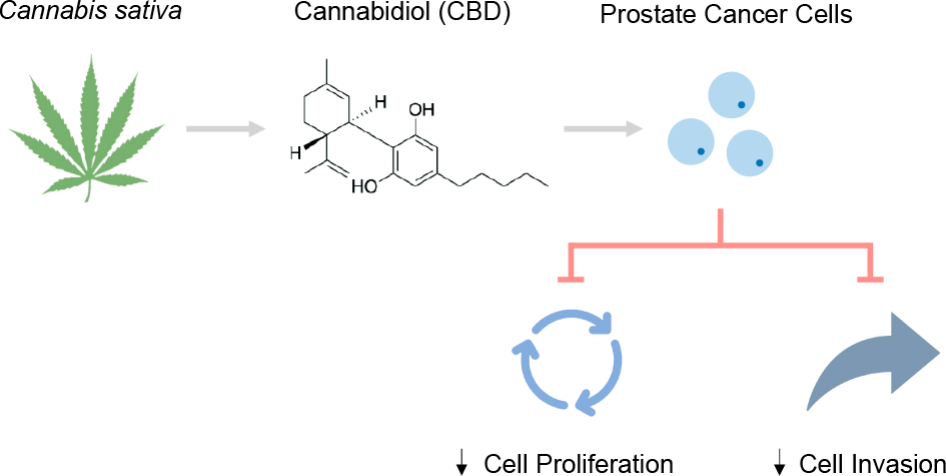

Prostate cancer is the fifth leading cause of cancer death in men, responsible for over 375,000 deaths in 2020. Novel therapeutic strategies are needed to improve outcomes. Cannabinoids, chemical components of the cannabis plant, are a possible solution. Preclinical evidence demonstrates that cannabinoids can modulate several cancer hallmarks of many tumor types. However, the therapeutic potential of cannabinoids in prostate cancer has not yet been fully explored. The aim of this study was to investigate the antiproliferative and anti-invasive properties of cannabidiol (CBD) in prostate cancer cells *in vitro*. CBD inhibited cell viability and proliferation, accompanied by reduced expression of key cell cycle proteins, specifically cyclin D3 and cyclin-dependent kinases CDK2, CDK4, and CDK1, and inhibition of AKT phosphorylation. The effects of CBD on cell viability were not blocked by cannabinoid receptor antagonists, a transient receptor potential vanilloid 1 (TRPV1) channel blocker, or an agonist of the G-protein-coupled receptor GPR55, suggesting that CBD acts independently of these targets in prostate cancer cells. Furthermore, CBD reduced the invasiveness of highly metastatic PC-3 cells and increased protein expression of E-cadherin. The ability of CBD to inhibit prostate cancer cell proliferation and invasiveness suggests that CBD may have potential as a future chemotherapeutic agent.

Prostate cancer is the fifth leading cause of cancer death in men, resulting in an estimated 375,000 deaths worldwide in 2020.^1^ Localized prostate cancer is quite treatable, with a 5-year survival rate close to 100%. However, when prostate cancer progresses to metastatic disease, the 5-year survival rate drops to just 30% (https://www.cancer.org/research/cancer-facts-statistics). Androgen deprivation therapy, the standard of care for metastatic prostate cancer, is initially effective in most patients. However, treatment resistance inevitably emerges over time with the development of castration-resistant disease, which is currently considered incurable. Therefore, novel therapeutic strategies are urgently needed to improve clinical outcomes for patients with metastatic and castration-resistant prostate cancer.

Cannabinoids, chemical compounds extracted from cannabis plants, pose a potential solution. Cannabidiol (CBD) may be a particularly attractive therapeutic option due to its lack of intoxicating properties. CBD-based medicines have already proven safe and effective for the treatment of various medical conditions, including epilepsy and multiple sclerosis.^2^ Furthermore, a growing body of *in vitro* and *in vivo* evidence demonstrates the anticancer potential of CBD. Studies on various cancer types show that CBD can modulate many key processes involved in cancer development and progression, including cell proliferation, apoptosis, cell migration and invasion, and angiogenesis.^3−7^ However, the chemotherapeutic potential of CBD in prostate cancer has not been extensively investigated.

Some preclinical evidence indicates the chemotherapeutic potential of CBD in prostate cancer cells. de Petrocellis et al. showed that CBD induced cell death through increased apoptosis in androgen-sensitive LNCaP cells, partly mediated through antagonism of the ion channel transient receptor potential metastatin 8 (TRPM8), which is involved in androgen receptor (AR)-dependent prostate cancer cell survival.^8^ The CBD-induced apoptosis was accompanied by increased reactive oxygen species production, increased levels of p53 upregulated modulator of apoptosis (PUMA), C/EBP homologous protein (CHOP), and intracellular calcium, activation of p53, and downregulation of AR. Similarly, Sreevalsan et al. showed that CBD induced apoptosis in LNCaP cells by increasing phosphatase expression, leading to cleavage of poly ADP-ribose polymerase (PARP) and caspase-3.^9^ Sharma et al. reported that a whole plant extract containing CBD and other cannabinoids inhibited LNCaP cell viability, accompanied by reduced expression of AR and prostate-specific antigen (PSA).^10^ Additionally, Motadi et al. reported that CBD reduced cell viability and increased caspase activity in hormone-resistant PC-3 cells, accompanied by increased expression of p53 and Bax.^11^ More recently, Mahmoud et al. investigated the role of metabolic signaling in the induction of cell death by CBD. They reported that CBD reduced cell viability and increased caspase activity in both hormone-naïve and hormone-resistant transgenic adenocarcinoma mouse prostate (TRAMP) cell line models, effects that were associated with increased glycolytic capacity, inhibition of oxidative phosphorylation and ATP production, and altered expressed of genes and proteins involved in regulating mitochondrial activity.^12^ While several studies have explored the induction of cell death by CBD in prostate cancer, the effect of cannabinoids on other cancer-related processes such as cell proliferation and cell invasion has not been thoroughly assessed.

Cell proliferation and cell invasion are two fundamental hallmarks of cancer cells. Therefore, compounds with antiproliferative and anti-invasive properties are potentially effective treatment options for cancer, particularly for advanced or metastatic disease. Substantial evidence indicates that CBD inhibits cell proliferation in many cancer types.^3,5,13−17^ Common underlying mechanisms include modulation of key cell cycle regulators, such as cyclins and cyclin-dependent kinases (CDKs), and inhibition of protein phosphorylation. For example, CBD induced cell cycle arrest in pancreatic cancer cells by inhibiting extracellular signal-related kinase (ERK) phosphorylation and reducing cyclin D expression.^15^ In gastric cancer cells, CBD reduced proliferation by modulating p21 and p53 expression, leading to inhibition of CDK2/cyclin E complex formation.^5^ In breast cancer and multiple myeloma cells, CBD inhibited proliferation through reduced phosphorylation and activation of ERK and AKT.^3,18^ In prostate cancer, studies have reported antiproliferative effects of synthetic cannabinoids. For instance, WIN55,212-2 (WIN), a synthetic cannabinoid receptor agonist, induced cell cycle arrest in prostate cancer cells, accompanied by increased p27 expression and reduced expression of CDK4 and phosphorylated retinoblastoma protein.^19^ Investigations into the *in vivo* antitumor activity of cannabinoids in prostate cancer have also focused primarily on synthetic cannabinoids.^20^ For example, WIN inhibited tumor growth in PC-3, DU145, and LNCaP xenograft mouse models.^19,21^ Meanwhile, limited evidence exists regarding the antiproliferative effects of plant-derived cannabinoids in prostate cancer. However, one study by De Petrocellis et al. reported that a CBD-rich cannabis plant extract inhibited the growth of LNCaP xenografts, suggesting that cytostatic and cytotoxic effects of plant-derived cannabinoids can translate to animal models of prostate cancer.^8^

Preclinical studies in several cancer types have reported antimetastatic properties of CBD. For instance, CBD inhibits cell migration and invasion in preclinical models of breast cancer, lung cancer, and cervical cancer, accompanied by reduced secretion of matrix metalloproteases and inhibition of the epithelial mesenchymal transition.^3,6,22,23^ Notably, numerous studies have also demonstrated antimetastatic activity of CBD *in vivo*.^23^ For example, CBD reduced the size and number of metastatic foci in mouse models of breast cancer and lung cancer.^3,24,25^ In prostate cancer, Pietrevito et al. assessed the effects of cannabinoids on the activity of stromal cells in the tumor microenvironment and showed that WIN reduced the invasiveness of cancer-associated fibroblasts (CAFs) and reduced the invasiveness of PC-3 cells subjected to conditioned media from the CAFs.^26^ However, the antimetastatic potential of plant-derived cannabinoids such as CBD in prostate cancer and the anti-invasive effects of cannabinoids in prostate tumor cells remain largely unexplored.

The aim of this study was to investigate the phenotypic effects and underlying mechanisms of action of CBD in cell line models of prostate cancer. We demonstrate that CBD inhibits prostate cancer cell proliferation, accompanied by the reduced expression of key cell cycle regulators and inhibition of AKT phosphorylation. Furthermore, CBD reduces the invasiveness of highly metastatic PC-3 cells. These findings suggest that CBD has potential as a future chemotherapeutic agent for prostate cancer.

## Results and Discussion

**Figure 1:**
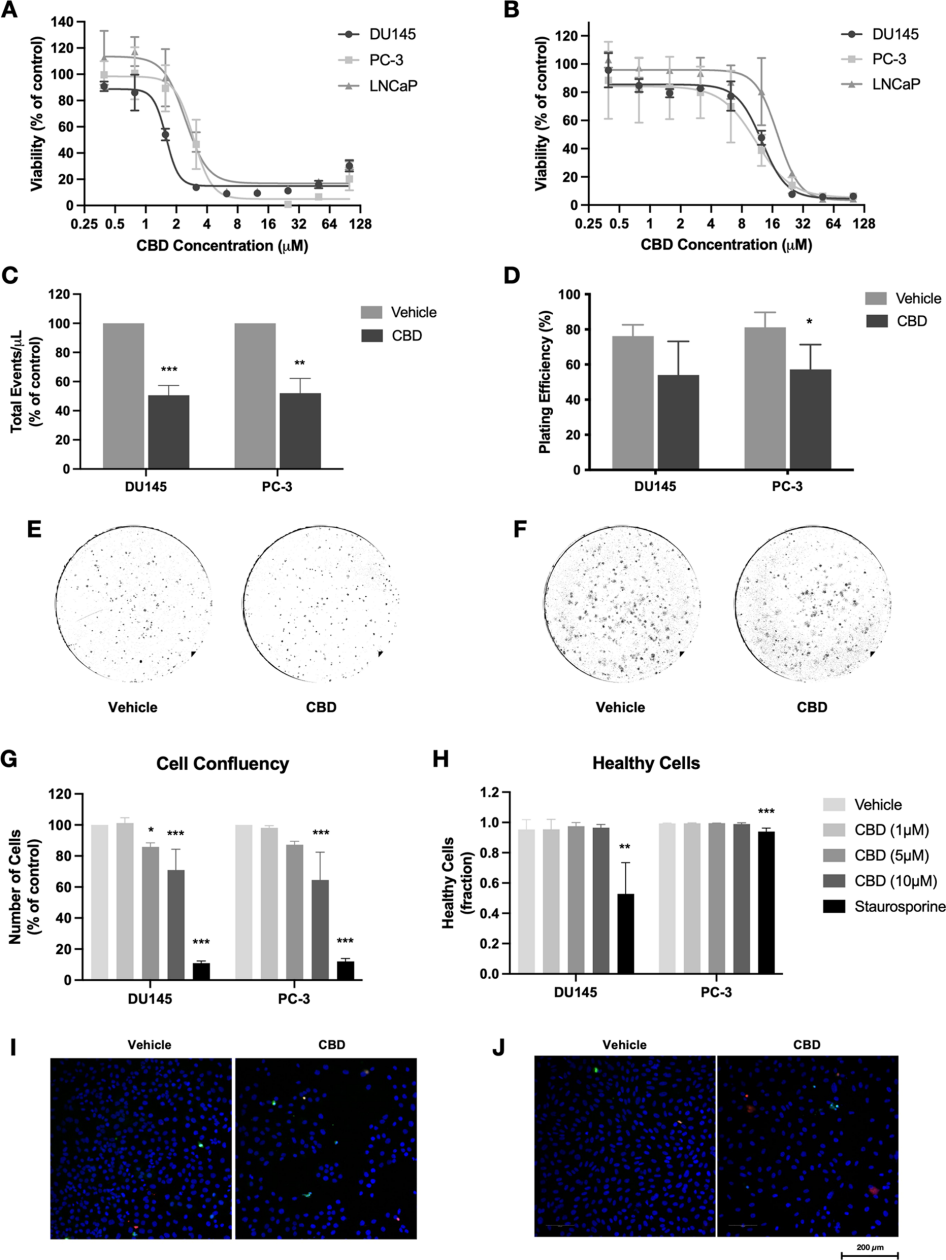
CBD reduces the viability, survival, and proliferation of prostate cancer cells. (A) Prostate cancer cells (DU145, PC-3, LNCaP) were treated with CBD (0–100 μM) for 72 h in the absence of serum. Cell viability was measured using the MTT assay. (B) Prostate cancer cells were treated with CBD in the presence of 10% FBS. Cell viability was measured using the MTT assay. (C) Androgen-insensitive cells (DU145, PC-3) were treated with IC_50_ doses of CBD for 48 h. Cell counts were determined using flow cytometry. (D) Cells were treated with IC_50_ doses of CBD for 48 h before reseeding without treatment. Colonies formed were counted after 7 days. (E) Representative images of colony formation in DU145 cells. (F) Representative images of colony formation in PC-3 cells. (G) Cells were treated with CBD (1, 5, 10 μM) for 72 h. Cell confluency was assessed by high-content fluorescence microscopy using Hoechst 33342 staining. Staurosporine was used as a positive control. (H) The fraction of healthy cells was measured using YO-PRO and propidium iodide (PI) staining. (I) Representative images of DU145 cells treated with vehicle or 10 μM CBD, stained with Hoechst 33342 (blue), YO-PRO (green), and PI (red). (J) Representative images of PC-3 cells treated with vehicle or 10 μM CBD. Data are represented as mean ± SD calculated from at least three independent experiments. *p < 0.05, **p < 0.01, ***p < 0.001 compared to the vehicle control for that cell line.

In this study, the phenotypic effects of CBD were assessed using androgen-sensitive (LNCaP) and androgen-insensitive (DU145, PC-3) prostate cancer cell lines. The MTT assay was used to measure cell viability following 72 h treatment with various doses of CBD (0–100 μM). Under serum deprivation conditions, CBD significantly reduced the viability of all three cell lines, with IC_50_ values of 1.5 μM (DU145), 2.9 μM (PC-3), and 2.6 μM (LNCaP) (Figure 1A). Because these cell lines are routinely grown in media containing serum, we also tested the effect of CBD in the presence of serum, which has previously been shown to reduce the efficacy of cannabinoids in prostate cells.^8^ As expected, IC_50_ values were higher in cells grown with serum, specifically, 12.3 μM (DU145), 10.5 μM (PC-3), and 18.0 μM (LNCaP) (Figure 1B). Notably, under these conditions, the androgen-independent DU145 and PC-3 cell lines were more sensitive to CBD treatment than the androgen-dependent LNCaP cells. To further investigate the inhibition of DU145 and PC-3 cell viability by CBD, total cell numbers were assessed using flow cytometry. Treatment with an IC_50_ dose of CBD significantly reduced cell counts for both cell lines, indicating a possible inhibitory effect on cell proliferation (Figure 1C). Additionally, clonogenic assay analysis revealed that 48 h CBD pretreatment significantly reduced the number of PC-3 cell colonies formed after 7 days by approximately 25% (*p* = 0.03) (Figure 1D–F), indicating that CBD reduces the ability of the cells to survive and proliferate following treatment.

To better understand how CBD reduces the viability of prostate cells, fluorescence microscopy was used to assess the effects of various CBD doses on cell proliferation and apoptosis in DU145 and PC-3 cells. Cells were treated with CBD (1, 5, or 10 μM) in the presence of serum and assessed using high-content screening microscopy. After 72 h treatment, CBD significantly reduced DU145 cell confluency at doses of 5 μM (*p* = 0.02) or 10 μM (*p* < 0.001) and reduced PC-3 cell confluency at a 10 μM dose (*p* < 0.001) (Figure 1G), indicating inhibition of cell proliferation. CBD did not reduce nucleus area or circularity, both indicative of cell death, at either time point at any of the doses tested (Supplementary Figure S1). Similarly, CBD did not significantly increase YO-PRO or propidium iodide positivity or reduce the fraction of healthy cells in either cell line (Figure 1H, Figure S2). Representative images of CBD-treated cells are shown (Figure 1I,J). The above findings were supported by flow cytometry analysis indicating that 48 h CBD treatment induced no significant pro-apoptotic effect in DU145 or PC-3 cells (Supplementary Figure S3). In agreement with the current study, a previous study reported that treatment with CBD in the presence of serum did not increase caspase activity or TUNEL positivity in DU145 or PC-3 cells.^8^ The above findings suggest that in cells grown with serum CBD reduces prostate cancer cell viability primarily through inhibition of cell proliferation, rather than through induction of apoptosis.

**Figure 2:**
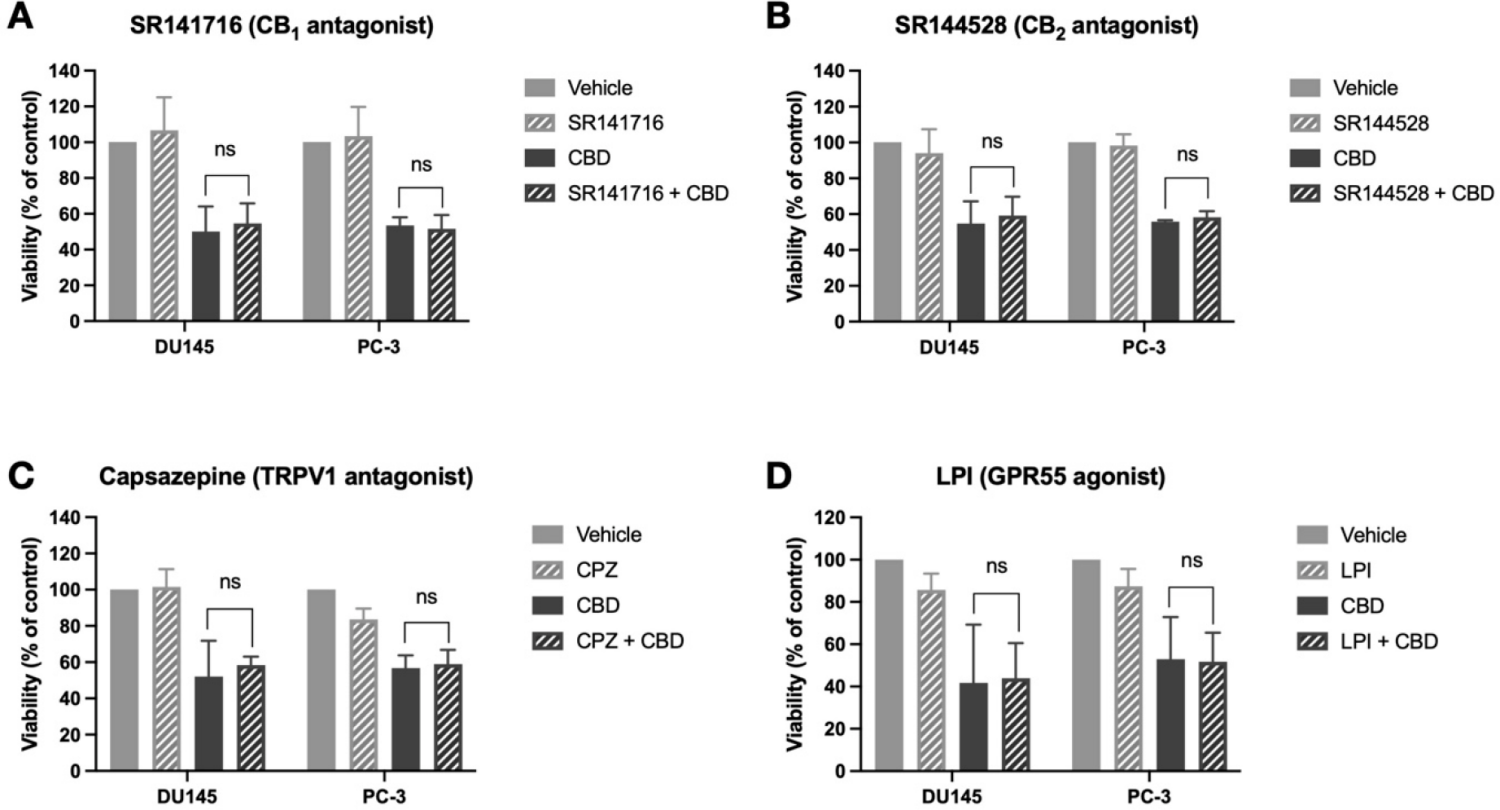
CBD reduces viability independently of CB_1_, CB_2_, TRPV1, or GPR55. (A) Prostate cancer cells (DU145, PC-3) were treated with SR141716 (CB_1_ antagonist) for 1 h before the addition of an IC_50_ dose of CBD for 72 h. Cell viability was determined by using the MTT assay. (B) Cells were treated with SR144528 (CB_2_ antagonist) for 1 h before the addition of an IC_50_ dose of CBD for 72 h. Cell viability was determined using the MTT assay. (C) Cells were treated with capsazepine (CPZ) (TRPV1 antagonist) for 1 h before the addition of an IC_50_ dose of CBD for 72 h. Cell viability was determined using the MTT assay. (D) Cells were treated with lysophosphatidylinositol (LPI) (GPR55 agonist) for 1 h before the addition of an IC_50_ dose of CBD for 72 h. Cell viability was determined using the MTT assay. Data are represented as mean ± SD calculated from at least three independent experiments. *p < 0.05 compared to cells treated with CBD alone.

One of the major challenges in elucidating cannabinoid mechanisms of action is identifying the receptor targets that mediate cannabinoid phenotypic effects. Cannabinoids play roles in many physiological processes and can modulate the activity of a wide range of receptors and targets.^27^ Common targets of cannabinoids in cancer cells include the major cannabinoid receptors, CB_1_ and CB_2_, the transient receptor potential vanilloid 1 (TRPV1) ion channel, and the putative novel cannabinoid receptor GPR55.^15,22,28,29^ All the above targets have previously been demonstrated to be present in prostate cancer cells.^8,30−32^ To identify the receptor targets of CBD, prostate cancer cells were pretreated with selective antagonists/agonists of common cannabinoid receptor targets, before assessing the effects of CBD on cell viability. Specifically, cells were pretreated with SR141716 (CB_1_ antagonist), SR144528 (CB_2_ antagonist), capsazepine (TRPV1 channel antagonist), or lysophosphatidylinositol (LPI) (GPR55 agonist), before 72 h treatment with an IC_50_ dose of CBD. However, no significant difference in viability was observed in cells pretreated with the any of the antagonists/agonists compared to cells treated with CBD alone, in any of the cell lines (Figure 2). These results suggest that the CBD-induced reduction in prostate cancer cell viability does not require the activation of CB_1_, CB_2_, or TRPV1 or the antagonism of GPR55. In agreement with these findings, Mahmoud et al. recently reported that the effect of CBD on cell viability was not blocked by antagonists of CB_1_, CB_2_, or TRPV1 in the TRAMP cell line model of prostate cancer.^12^ However, cannabinoids can modulate the activity of cannabinoid receptors through mechanisms other than direct activation or antagonism. For example, CBD can act as a negative allosteric modulator of CB_1_, altering receptor conformation and ligand-binding activity.^33,34^ Some studies report that CBD acts as an inverse agonist at CB_2_, while others identify CBD as a partial CB_2_ agonist.^34,35^ Knocking down these targets using siRNA could provide insight into whether CBD is indirectly modulating the activity of these receptors. Alternatively, CBD may reduce the viability of prostate cancer cells primarily by interacting with many other cannabinoid targets. Previous studies have shown that cannabinoids can alter cancer-related processes through interactions with the transcription factor peroxisome proliferator activated receptor gamma (PPARγ), the mitochondrial protein voltage-dependent anion-selective channel 1 (VDAC1), the ion channels TRPM8 and transient receptor potential ankyrin 1 (TRPA1), serotonin receptors, and steroid receptors, among many other targets.^12,36−39^

**Figure 3:**
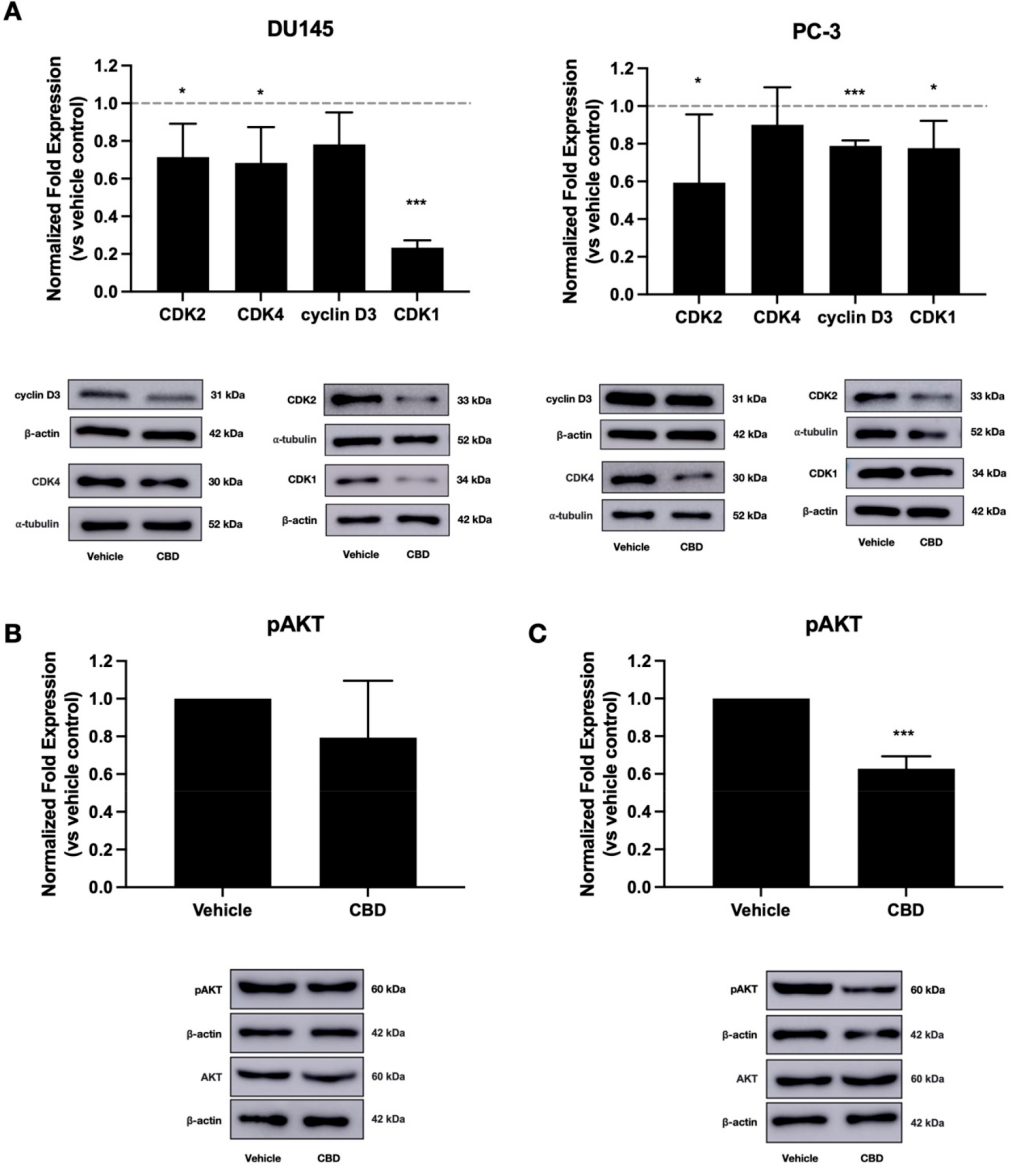
CBD alters the expression of proteins involved in cell proliferation. (A) Prostate cancer cells (DU145, PC-3) were treated with IC_50_ doses of CBD for 48 h. Expression of CDK2, CDK4, cyclin D3, and CDK1 was measured using Western blotting. (B) PC-3 cells were treated with an IC_50_ dose of CBD for 48 h. AKT phosphorylation was measured by using Western blotting. (C) DU145 cells were treated with an IC_50_ dose of CBD for 48 h. AKT phosphorylation was measured using Western blotting. Images of representative blots are shown. Data are represented as mean ± SD calculated from at least three independent experiments. *p < 0.05, ***p < 0.001 compared to the vehicle control.

While cannabinoid mechanisms have been extensively studied in certain cancer types, for example, glioblastoma and breast cancer, the mechanisms driving the phenotypic effects of cannabinoids in prostate cancer are not fully understood. Increased cell proliferation in cancer is associated with increased activity of cyclins and CDKs, which drive cell cycle progression.^40^ Inhibition of cell cycle progression is a useful therapeutic strategy in cancer, and several CDK inhibitors have been approved for cancer treatment.^41^ Some existing studies show that cannabinoids can reduce the levels of expression of cyclins and CDKs in cancer cells. In multiple myeloma and pancreatic cancer, for example, inhibition of cell proliferation by CBD was accompanied by reduced expression of cyclin D.^15,18^ In gastric cancer, CBD inhibited the formation of the CDK2/cyclin E complex, which drives cell cycle progression.^5^ Having shown that CBD inhibits prostate cancer cell proliferation, we investigated whether CBD treatment modulates the expression of cyclins and CDKs in these cells. To determine whether CBD induces G1/S phase cell cycle arrest in prostate cancer cells, we measured the expression of CDK2, CDK4, and cyclin D3, which promote the G1/S phase cell cycle transition. In DU145 cells, CBD significantly reduced the expression of CDK2 (*p* = 0.049) and CDK4 (*p* = 0.04) (Figure 3A). Additionally, CBD reduced the level of cyclin D3 expression by approximately 20%, though this was not statistically significant. Furthermore, CBD significantly reduced the expression of cyclin D3 (*p* = 0.0002) and CDK2 (*p* = 0.04) in PC-3 cells (Figure 3A). These results indicate that CBD may induce G1/S phase cell cycle arrest in prostate cancer cells by reducing the level of expression of key proteins that drive the G1/S phase transition. Our results complement previous findings that CBD increases the expression of the CDK inhibitors p21 and p27^kip^ in prostate cancer cells.^8^

To assess whether CBD induces cell cycle arrest at the G2/M checkpoint, we also measured the expression of CDK1, a key regulator of cell cycle progression primarily involved in the G2/M phase transition, and CDK7, which also drives G2/M phase progression. CBD significantly reduced CDK1 expression in both DU145 (*p* < 0.0001) and PC-3 (*p* = 0.02) cells, with a particularly strong effect in DU145 cells (Figure 3A). CBD had no significant effect on CDK7 expression in either cell line (Supplementary Figure S4). These results provide evidence that CBD induces G2/M cell cycle arrest through downregulation of CDK1. Some evidence suggests that the psychoactive plant-derived cannabinoid THC inhibits breast cancer cell proliferation by reducing the expression of CDK1.^42^ Additionally, one recent study showed that a synthetic CBD analogue inhibited CDK1 mRNA expression in a model of cardiac fibrosis.^43^ However, to the best of our knowledge, this study provides the first evidence that CBD reduces CDK1 expression in cancer. Together, the above results indicate that CBD downregulates key proteins that drive cell cycle progression through both the G1/S and G2/M checkpoints, further supporting our findings that CBD inhibits prostate cancer cell proliferation.

Phosphorylation and activation of the protein kinase AKT promotes cancer cell proliferation, survival, and invasiveness.^44−46^ AKT hyperphosphorylation is a common feature of prostate cancer, with increased AKT activity observed in 50% of prostate cancers.^47^ Previous studies show that CBD reduces AKT phosphorylation in glioma, multiple myeloma, leukemia, and breast cancer, accompanied by reduced cell proliferation, migration, and invasion, and increased apoptosis.^3,13,18,48,49^ Here, we used Western blotting to assess the effect of CBD treatment on AKT signaling. CBD had no significant effect on AKT phosphorylation in PC-3 cells (Figure 3B). However, CBD significantly reduced AKT phosphorylation by approximately 40% in DU145 cells (*p* = 0.0006) (Figure 3C), suggesting that inhibition of AKT phosphorylation may contribute to the CBD-induced reduction in the expression of cell cycle proteins in these cells. These results support a recent report that CBD can inhibit AKT phosphorylation in the TRAMP cell line model of prostate cancer.^12^ Our results suggest that in DU145 cells CBD may reduce cell viability by inhibiting the phosphorylation and activation of AKT, leading to downregulation of the cell cycle proteins CDK1, CDK2, and CDK4, induction of cell cycle arrest, and inhibition of cell proliferation. Alternatively, reduced AKT phosphorylation may occur downstream of the observed changes in cyclin and CDK expression.

**Figure 4:**
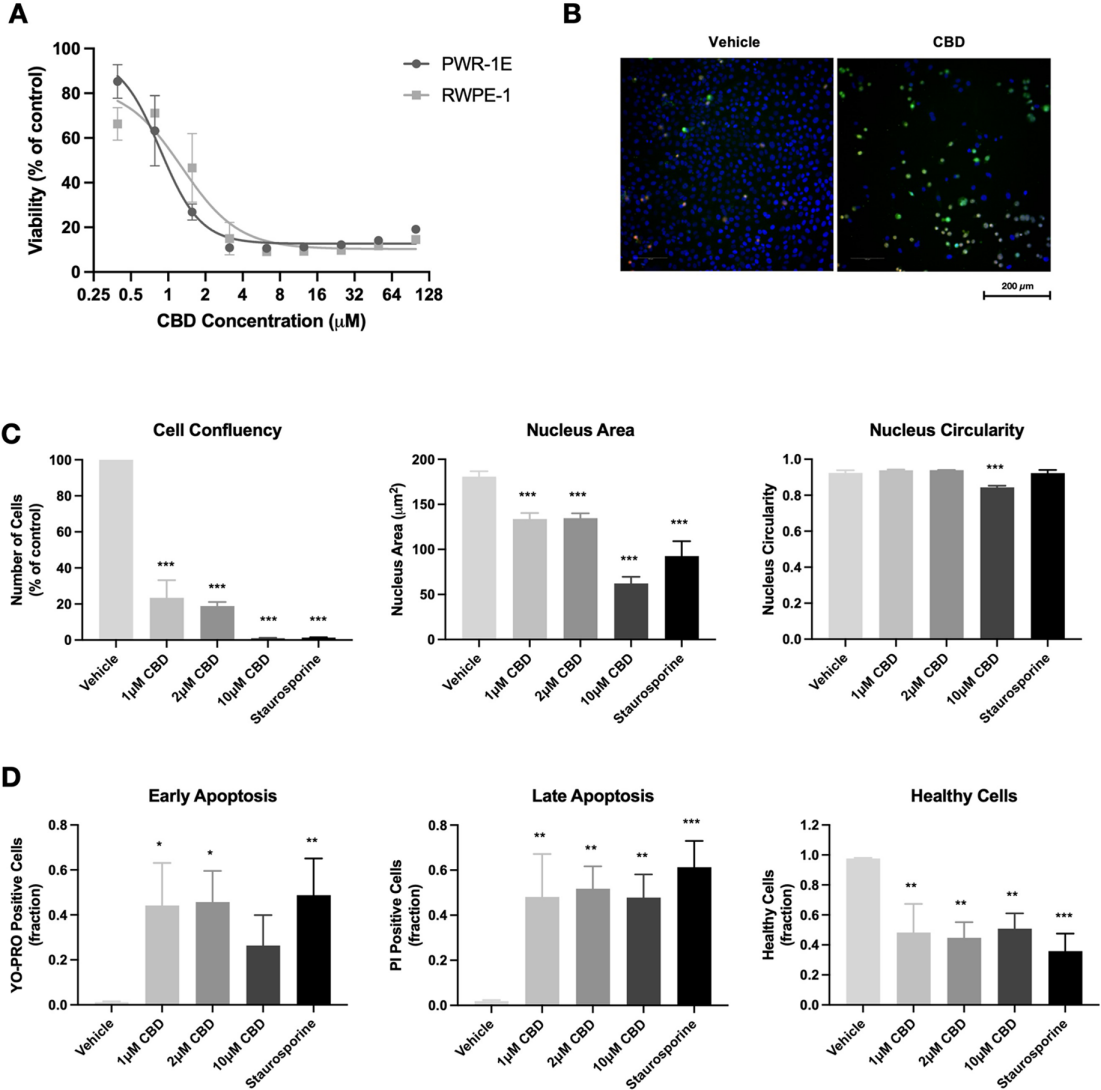
CBD induces apoptosis in noncancerous prostate cells. (A) Noncancerous prostate epithelial cells (PWR-1E, RWPE-1) were treated with CBD (0–100 μM) for 72 h in the absence of serum. Cell viability was measured using the MTT assay. (B) Representative images of PWR-1E cells treated with vehicle or 1 μM CBD for 72 h, stained with Hoechst 33342 (blue), YO-PRO (green), and PI (red). (C) The effect of CBD on PWR-1E cell confluency, nucleus area, and nucleus circularity was assessed using high-content fluorescence microscopy. (D) Fractions of early apoptotic, late apoptotic, and healthy PWR-1E cells were measured by fluorescence microscopy, using YO-PRO and PI staining. Data are represented as mean ± SD calculated from at least three independent experiments. *p < 0.05, **p < 0.01, ***p < 0.001 compared to the vehicle control.

A key finding from this study was the effect exerted by CBD on noncancerous prostate epithelial cells. To determine whether the effects of CBD on viability were cancer cell-specific, MTT analysis was conducted using two noncancerous androgen-sensitive prostate epithelial cell lines, PWR-1E and RWPE-1. Interestingly, the noncancerous cell lines were slightly more sensitive to CBD treatment than the cancer cell lines, with IC_50_ values of 0.9 μM (PWR-1E) and 1.1 μM (RWPE-1) (Figure 4A), suggesting that the effects of CBD on viability are not specific to cancer cells. Fluorescence microscopy was used to determine whether the reduced cell viability occurred through an increased level of cell death or inhibition of cell proliferation. PWR-1E cells were treated with CBD (1, 2, or 10 μM) in the absence of serum. CBD significantly reduced the cell confluency at all doses tested. CBD also reduced nucleus area (*p* < 0.001) and circularity (*p* < 0.001), indicative of increased cell death (Figure 4B,C). Furthermore, CBD increased YO-PRO and propidium iodide positivity and reduced the fraction of healthy cells (Figure 4B,D). These results indicate that CBD induces cell death through increased apoptosis in noncancerous PWR-1E cells. The above findings contrast with previous studies reporting that CBD reduced cell viability in cell line models of colon cancer, breast cancer, and head and neck squamous cell carcinoma, without affecting the viability of corresponding noncancerous cell lines.^4,7,29,50^ Furthermore, Sharma et al. reported that CBD inhibited the viability of prostate cancer cells with no significant effect observed in the prostate epithelial cell lines BPH-1 and PNT1B.^10^ Notably, both cell lines used in that study were grown with serum, which is likely to reduce the efficacy of CBD. Furthermore, all the above experiments were conducted following a shorter 24 h CBD treatment, and it is possible that a reduction in viability would be seen with longer treatments. In agreement with the current study, Deng et al. reported that 72 h CBD treatment reduced viability at similar potencies in glioblastoma cells and in noncancerous neural progenitor cells.^51^ Similarly, a recent study in cholangiocarcinoma showed that CBD effects were not specific to cancer cells.^52^ It should be noted that the current study used immortalized cell lines, which are artificially transformed to proliferate indefinitely, a process known to alter cellular properties including differentiation, DNA damage response, and chromosome structure.^53,54^ Therefore, the observed cytotoxic effects may not necessarily translate to similar effects in true normal and healthy prostate cells. In fact, the doses used in the current study are well within the range that has been reported safe and well-tolerated *in vivo.* Several studies report that CBD doses up to 1500 mg/day are safe and well-tolerated in humans.^55^ Moreover, cannabis-based medicines are currently approved for the treatment of various medical conditions and display low levels of toxicity. However, our findings suggest that the effects of CBD are not cancer cell-specific. A deeper investigation into receptor targets of CBD and effects of CBD on intracellular signaling pathways may provide greater insight into potential off-target effects and toxicity.

**Figure 5:**
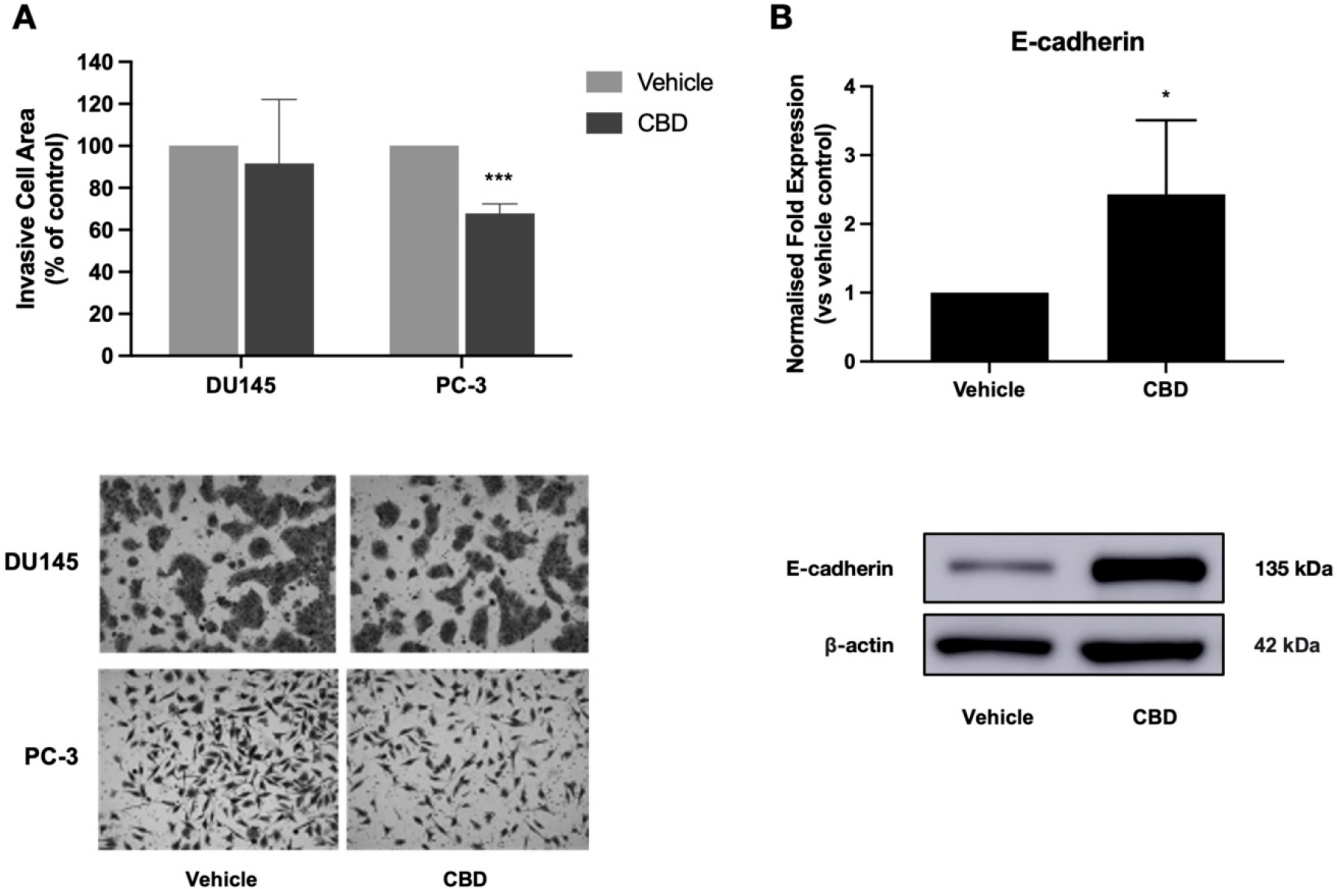
CBD reduces the extent of invasiveness of PC-3 cells. (A) Prostate cancer cells (DU145, PC-3) were treated with a noncytotoxic dose of CBD for 48 h. Cell invasion was assessed using the Transwell assay. Representative images of invasive cells are shown. (B) PC-3 cells were treated with a noncytotoxic dose of CBD for 48 h. Expression of E-cadherin was measured using Western blotting. Images of representative blots are shown. Data are represented as mean ± SD calculated from at least three independent experiments. *p < 0.05, ***p < 0.001 compared to the vehicle control.

Activation of cell invasion and metastasis is another crucial step in cancer progression, and metastasis is the cause of approximately 90% of cancer deaths.^56^ Thus, agents that can inhibit metastasis could play an important role in cancer treatment. Previous studies have shown that cannabinoids reduce cell invasion in several cancer types, including breast cancer, lung cancer, glioblastoma, and head and neck squamous cell carcinoma.^3,6,50,57−59^ In breast cancer, inhibition of cell invasion by CBD was accompanied by reduced secretion of the matrix metalloproteases MMP-2 and MMP-9.^3^ An additional study reported that CBD was capable of reverting the invasive mesenchymal phenotype of breast cancer cells to a noninvasive epithelial phenotype, accompanied by increased expression of the adherens junction protein E-cadherin.^6^ Here, we assessed the antimetastatic potential of CBD in prostate cancer. Treatment with a noncytotoxic dose of CBD significantly reduced the invasiveness of highly metastatic PC-3 cells by approximately 30% (*p* = 0.0003) (Figure 5A). CBD had no significant effect on DU145 cell invasion. To investigate the mechanisms underlying the observed CBD-induced reduction in PC-3 cell invasion, we measured the secretion of matrix metalloproteases and the expression of E-cadherin. CBD treatment did not alter the secretion of MMP-1, MMP-3, or MMP-9 (Supplementary Figure S5). Interestingly, treatment with a noncytotoxic dose of CBD induced a greater than 2-fold increase in E-cadherin expression in PC-3 cells (*p* = 0.0374) (Figure 5B). These results indicate that the CBD-induced reduction in PC-3 invasiveness may occur through increased expression of the cell adhesion protein E-cadherin, suggesting that CBD may promote a noninvasive epithelial phenotype in prostate cancer cells. To our knowledge, these findings provide the first evidence that plant-derived cannabinoids have anti-invasive activity in prostate tumor cells.

It is important to note that the above phenotypic screening was conducted using artificial 2D cell line models. Further assessment of the effects of CBD in more physiologically relevant models, for example, 3D cell culture models or animal models, will determine the likelihood of the observed phenotypic effects translating to therapeutic benefits in a clinical setting.

In conclusion, this study provides novel insights into the phenotypic effects and underlying mechanisms of action of the plant-derived cannabinoid CBD in prostate cancer. CBD inhibits the viability, proliferation, survival, and invasiveness of prostate cancer cells. CBD-induced inhibition of proliferation was associated with reduced expression of the cell cycle regulators cyclin D3, CDK4, CDK2, and CDK1 and reduced phosphorylation of the protein kinase AKT. The anti-invasive effects of CBD were accompanied by increased expression of E-cadherin. Further research is needed to identify the receptor target(s) of CBD in prostate cancer cells, to gain a deeper understanding of the mechanisms of action, and to investigate the effects of CBD on noncancerous cells. Additionally, testing of CBD in more biologically relevant models will determine whether the promising effects observed *in vitro* are likely to translate to therapeutic benefits. Overall, our findings indicate that CBD may have potential as a future chemotherapeutic agent in prostate cancer.

## Experimental Section

### General Experimental Procedures

Androgen-insensitive (DU145 ACC 261, PC-3 ACC 465) and androgen-sensitive (LNCaP ACC 256) human prostate cancer cell lines were purchased from the German Collection of Microorganisms and Cell Cultures (DSMZ). Noncancerous human prostate epithelial cell lines (PWR-1E CRL-11611, RWPE-1, CRL-11609) were purchased from the American Type Culture Collection (ATCC). DU145, PC-3, and LNCaP cells were cultured using RPMI 1640 medium with GlutaMAX (Gibco), supplemented with 10% fetal bovine serum (FBS) (Gibco). PWR-1E and RWPE-1 cells were cultured using keratinocyte serum-free medium supplemented with l-glutamine, bovine pituitary extract (50 mg/L), and epidermal growth factor (5 μg/L) (Gibco). Cells were maintained in a humidified 5% CO_2_ incubator at 37 °C.

CBD was provided by GreenLight Pharmaceuticals. CBD purity of >99.7% was confirmed by convergence chromatography. SR141716 (CB_1_ antagonist), SR144528 (CB_2_ antagonist), capsazepine (TRPV1 antagonist), LPI (GPR55 agonist), and staurosporine (apoptosis positive control) were purchased from Sigma-Aldrich. CBD, SR141716, SR144528, and capsazepine were dissolved by using dimethyl sulfoxide (DMSO) (PanReac AppliChem). LPI was dissolved using sterile dH_2_O. All drug compounds were stored according to the manufacturer’s instructions.

### MTT Assay

Cells were seeded in 96-well plates at optimized densities of 1 × 10^3^ cells per well (DU145, PC-3), 6 × 10^3^ cells per well (LNCaP), 2 × 10^3^ cells per well (PWR-1E), or 4.5 × 10^3^ cells per well (RWPE-1). Cells were allowed to adhere for 24 h before drug treatments were applied. For serum deprivation, treatments were applied in a serum-free medium. For antagonist experiments, cells were pretreated with antagonists for 1 h before the addition of CBD. After drug treatment, 5 mg/mL thiazolyl blue tetrazolium bromide (MTT) reagent (Sigma-Aldrich) was added to each well, and the plates were incubated at 37 °C. After 3 h of incubation, the culture medium and MTT reagent were discarded, and the formazan crystals were dissolved using DMSO. Absorbance was measured at 570 nm using a CLARIOstar microplate reader (BMG Labtech). The percentage viability was calculated relative to the vehicle control.

### Clonogenic Assay

After 24 h adhesion, cells were treated for 48 h, then detached and reseeded in six-well plates at low cell densities of 250 cells per well (DU145) or 500 cells per well (PC-3), without treatment. Cells were maintained at 37 °C and 5% CO_2_ for 7 days, with the culture medium replaced every 2–3 days. After 7 days, the culture medium was removed, and the cells were washed with Dulbecco’s phosphate-buffered saline (PBS) (Gibco). The colonies formed were fixed and stained using a glutaraldehyde/crystal violet solution (3.6 mL glutaraldehyde, 25% aqueous solution (Thermo Fisher Scientific) + 3.26 mL 2.3% crystal violet solution (Sigma-Aldrich) + 8.14 mL dH_2_O). Colonies were counted using ImageJ image analysis software.

### Transwell Invasion Assay

Falcon 8.0 μm pore cell culture inserts (Analab) were added to a 24-well plate, coated with 100 μL of 1 mg/mL extracellular matrix (ECM) (Sigma-Aldrich), and incubated at 37 °C for 1 h to allow gel polymerization. Cells were seeded in serum-free medium containing CBD or vehicle in the upper compartment of the insert at a density of 5 × 10^4^ cells per well. To provide a chemotactic gradient, 500 μL of complete medium containing 10% FBS was added to the well underneath. After 48 h of treatment, the medium was discarded, and noninvasive cells were removed from the upper compartment using a cotton swab. Invasive cells were stained for 10 min using a 0.23% crystal violet solution. Inserts were washed and allowed to dry overnight. Images of invasive cells were captured on a Nikon Eclipse E600 microscope using a 10× objective. The invasive cell area was measured using ImageJ software.

### Fluorescence Microscopy

Cells were seeded in black-walled PhenoPlate 96-well microplates (PerkinElmer) at densities of 2 × 10^3^ cells per well. After drug treatment, cells were stained for 30 min using 1:1000 YO-PRO-1 (Invitrogen), 1:1000 propidium iodide (Invitrogen), and 1:5000 Hoechst 33342 (Sigma-Aldrich) in culture medium. Cells were imaged using an Opera Phenix high-content screening system (PerkinElmer) fitted with a 63×/1.15 NA water immersion objective. Images were analyzed using Harmony v4.8 high-content imaging and analysis software (PerkinElmer).

### Flow Cytometry

Cells were seeded in six-well plates at densities of 4 × 10^4^ cells per well (DU145) or 3 × 10^4^ cells per well (PC-3). After drug treatment, supernatants were collected, and the remaining adherent cells were detached using trypsin. The supernatant and detached cell suspension were combined and centrifuged, and the resulting cell pellets were resuspended in ice-cold PBS containing YO-PRO (1:1000) and propidium iodide (1:1000). Samples were analyzed by using an Accuri C6 flow cytometer (BD Biosciences). Cells were identified by using forward scatter and side scatter gating. YO-PRO and propidium iodide staining were used to gate for live cells, early apoptotic cells, late apoptotic cells, and DNA fragmentation.

### Western Blotting

Primary antibodies against CDK2, CDK4, cyclin D3, CDK1, phosphorylated AKT, and total AKT were purchased from Cell Signaling Technology. E-cadherin primary antibody was purchased from BD Biosciences. β-Actin primary antibody was purchased from Sigma-Aldrich. α-Tubulin primary antibody was purchased from Abcam. Secondary antibodies were purchased from Cell Signaling Technology. Cells were seeded in six-well plates at a density of 5 × 10^4^ cells per well. After drug treatment, cells were lysed using ice-cold RIPA buffer (Sigma-Aldrich) with a protease phosphatase inhibitor (Thermo Fisher Scientific). Total protein levels were quantified by using the DC protein assay (Bio-Rad). SDS-PAGE was conducted using a final concentration of 40 μg of protein per lane. Following SDS-PAGE, proteins were transferred to PVDF membranes (Millipore). Membranes were blocked using 5% skimmed milk before overnight incubation with primary antibodies at 4 °C. After washing, membranes were incubated with complementary horseradish peroxidase (HRP)-conjugated secondary antibodies for 1 h at room temperature. Immobilon Forte Western HRP substrate (Millipore) was added to each membrane, and proteins were visualized using an Amersham Imager 600 (GE Healthcare). Densitometry analysis was conducted using ImageJ software. The density values for each protein were normalized to the corresponding loading control and then to the vehicle-treated samples.

### Statistical Analysis

Data were analyzed by using GraphPad Prism 8 data analysis software. IC_50_ values were determined using nonlinear regression. One-way ANOVA was used to compare the effects of a range of drug concentrations against the vehicle control. Student’s *t*-test was used to compare mean values between vehicle-treated and CBD-treated samples. *p*-Values < 0.05 were considered statistically significant. Data are expressed as the mean ± SD. All results are representative of at least three independent experiments.
