# Supplementary material for: Cannabidiol Inhibits the Proliferation and Invasiveness of Prostate Cancer Cells
Source: J Nat Prod. 2023 Sep 13;86(9):2151–61. doi: 10.1021/acs.jnatprod.3c00363 (PMC10521019; doi:10.1021/acs.jnatprod.3c00363)
Supplement: Supplementary file 1 — np3c00363_si_001.pdf [file np3c00363_si_001.pdf]

Supporting Information

Cannabidiol Inhibits the Proliferation and Invasiveness of Prostate Cancer Cells

Eve O'Reilly,[§,†] Karima Khalifa,[§,†] Joanne Cosgrave,[§,†] Haleema Azam,[†,‡] Maria Prencipe,[†,‡] Jeremy C. Simpson,[§] William M. Gallagher,[†,‡] Antoinette S. Perry[§,†]*

[§]UCD School of Biology and Environmental Science, University College Dublin, Ireland, [†]Cancer Biology and Therapeutics Laboratory, Conway Institute of Biomolecular and Biomedical Research, University College Dublin, Ireland, [‡]UCD School of Biomolecular and Biomedical Science, University College Dublin, Ireland

Table of Contents

Supplementary Figures

Figure S1. CBD does not affect nucleus area or circularity in prostate cancer cells...............3
Figure S2. 72h CBD treatment does not induce apoptosis in prostate cancer cells 4
Figure S3. 48h CBD treatment does not induce apoptosis in prostate cancer cells 5
Figure S4. CBD does not alter CDK7 expression in prostate cancer cells..............................6
Figure S5. CBD does not alter secretion of MMP-1, MMP-3, or MMP-9 by PC-3 cells 7

Figure S1. CBD does not affect nucleus area or circularity in prostate cancer cells

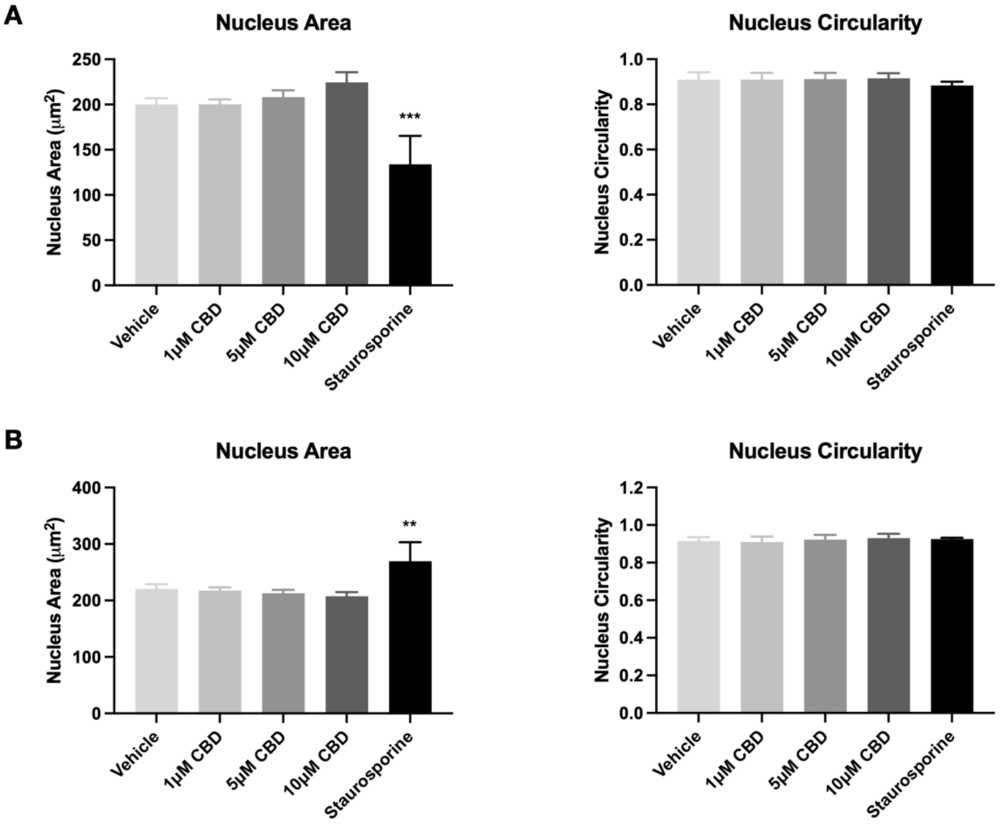

Supplementary Figure S1. CBD does not affect nucleus area or circularity in prostate cancer cells. (A) DU145 cells were treated with CBD (1μM, 5μM, 10μM) for 72h. Staurosporine was used as a positive control. Nucleus area and circularity were assessed by high-content fluorescence microscopy using Hoechst staining. (B) PC-3 cells were treated with CBD (1μM, 5μM, 10μM) or staurosporine for 72h. Nucleus area and circularity were assessed using high-content fluorescence microscopy. Data are represented as mean ± SD calculated from at least three independent experiments. *p<0.05 **p<0.01 ***p<0.001 compared to the vehicle control for that cell line.

Figure S2. 72h CBD treatment does not induce apoptosis in prostate cancer cells

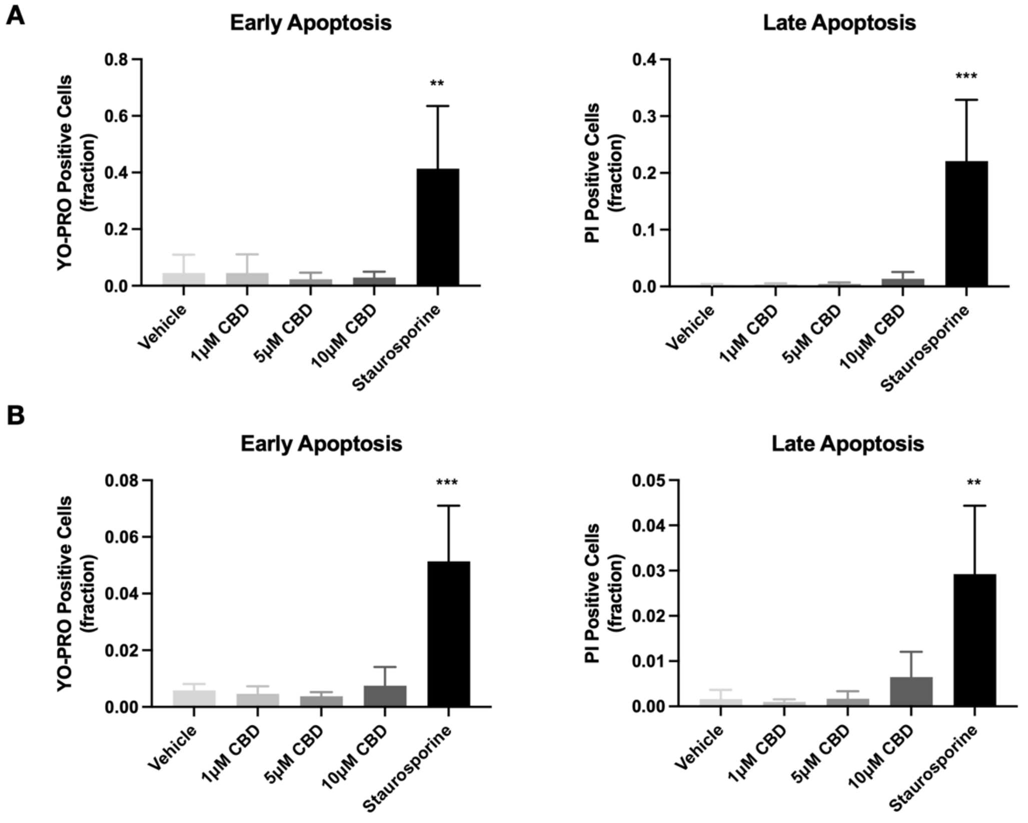

Supplementary Figure S2. 72h CBD treatment does not induce apoptosis in prostate cancer cells. (A) DU145 cells were treated with CBD (1μM, 5μM, 10μM) for 72h. Staurosporine was used as a positive control. Fractions of early apoptotic and late apoptotic cells were assessed by high-content fluorescence microscopy using YO-PRO and PI staining. (B) PC-3 cells were treated with CBD (1μM, 5μM, 10μM) or staurosporine for 72h. Fractions of apoptotic cells were assessed using high-content fluorescence microscopy. Data are represented as mean ± SD calculated from at least three independent experiments. *$p<0.05$ **$p<0.01$ ***$p<0.001$ compared to the vehicle control for that cell line.

Figure S3. 48h CBD treatment does not induce apoptosis in prostate cancer cells

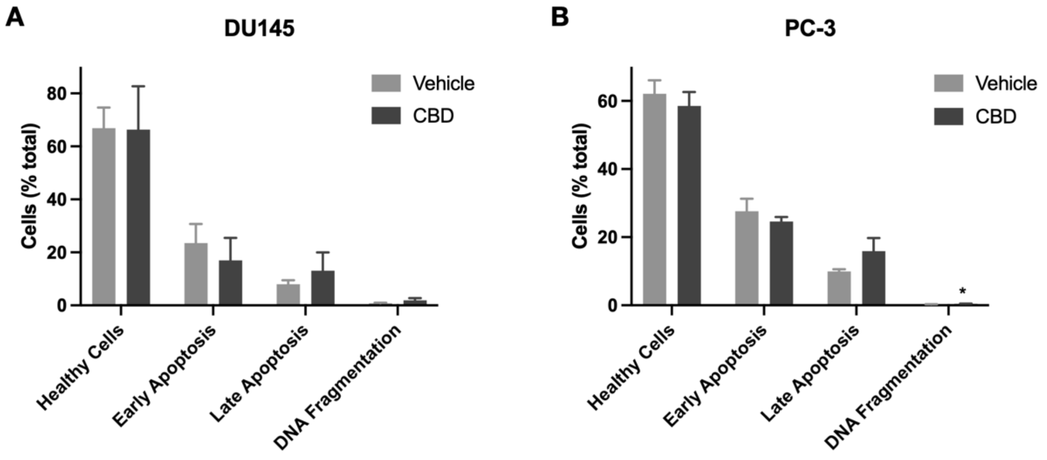

Supplementary Figure S3. 48h CBD treatment does not induce apoptosis in prostate cancer cells. (A) DU145 cells were treated with an IC_{50} dose of CBD for 48h. Fractions of early apoptotic, late apoptotic, and healthy cells were assessed by flow cytometry using YO-PRO and PI staining. (B) PC-3 cells were treated with an IC_{50} dose of CBD for 48h. Fractions of apoptotic and healthy cells were assessed using flow cytometry. Data are represented as mean ± SD calculated from at least three independent experiments. *p<0.05 compared to the vehicle control for that cell line.

Figure S4. CBD does not alter CDK7 expression in prostate cancer cells

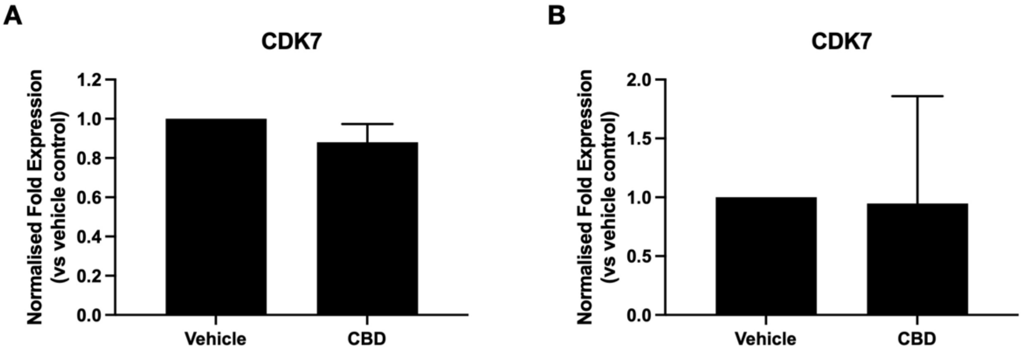

Supplementary Figure S4. CBD does not alter CDK7 expression in prostate cancer cells. (A) DU145 cells were treated with an IC_{50} dose of CBD for 48h. CDK7 expression was measured using Western blotting. (B) PC-3 cells were treated with an IC_{50} dose of CBD for 48h. CDK7 expression was measured using Western blotting. Data are represented as mean ± SD calculated from at least three independent experiments. *p<0.05 compared to the vehicle control for that cell line.

Figure S5. CBD does not alter secretion of MMP-1, MMP-3, or MMP-9 by PC-3 cells

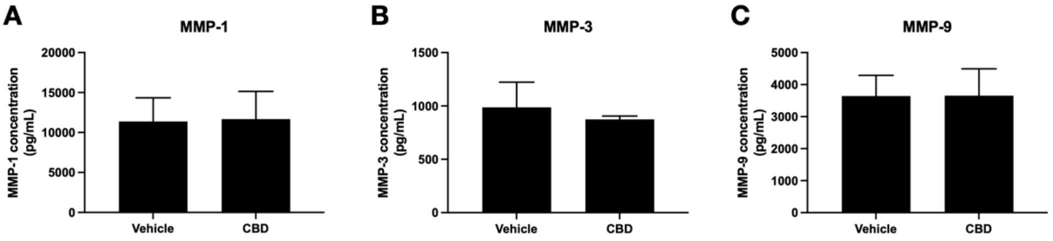

Supplementary Figure S5. CBD does not alter secretion of MMP-1, MMP-3, or MMP-9 by PC-3 cells. PC-3 cells were treated with a non-cytotoxic dose of CBD for 48h. (A) MMP-1 secretion was measured using ELISA. (B) MMP-3 secretion was measured using ELISA. (C) MMP-9 secretion was measured using ELISA. Data are represented as mean ± SD calculated from at least three independent experiments. *p<0.05 compared to the vehicle control.
